# Cell contractility arising from topography and shear flow determines human mesenchymal stem cell fate

**DOI:** 10.1038/srep20415

**Published:** 2016-02-16

**Authors:** Surabhi Sonam, Sharvari R. Sathe, Evelyn K.F. Yim, Michael P. Sheetz, Chwee Teck Lim

**Affiliations:** 1 Mechanobiology Institute, National University of Singapore, 117411, Singapore; 2 Department of Biomedical Engineering, National University of Singapore, 117583, Singapore; 3 Department of Surgery, National University of Singapore, 119228, Singapore; 4 Department of Biological Sciences, Columbia University, New York, 10027, USA; 5 Department of Mechanical Engineering, National University of Singapore, 117575, Singapore

## Abstract

Extracellular matrix (ECM) of the human Mesenchymal Stem Cells (MSCs) influences intracellular tension and is known to regulate stem cell fate. However, little is known about the physiological conditions in the bone marrow, where external forces such as fluid shear stress, apart from the physical characteristics of the ECM, influence stem cell response. Here, we hypothesize that substrate topography and fluid shear stress alter the cellular contractile forces, influence the genetic expression of the stem cells and hence alter their lineage. When fluid shear stress was applied, human MSCs with higher contractility (seeded on 1 μm wells) underwent osteogenesis, whereas those with lower contractility (seeded on 2 μm gratings) remained multipotent. Compared to human MSCs seeded on gratings, those seeded on wells exhibited altered alignment and an increase in the area and number of focal adhesions. When actomyosin contractility was inhibited, human MSCs did not exhibit differentiation, regardless of the topographical feature they were being cultured on. We conclude that the stresses generated by the applied fluid flow impinge on cell contractility to drive the stem cell differentiation via the contractility of the stem cells.

Due to the availability in adult tissues and differentiation potential, human MSCs have been exploited extensively for cell based therapies. However, limited knowledge of stem cell biology and impact of the cell microenvironment on them has hindered the usage of stem cells in cell based therapies.

Recent studies on the effects that biophysical cues have on MSCs reveal the importance of cell contractility in cell fate determination. Dominant influencers of cell fate include static forces generated by substrate microarchitecture, micropatterning and rigidity, as well as dynamic forces, such as fluid flow. Together, these forces influence the cell fate determination process by changing the extent of cell spreading, cell morphology, the arrangement of focal adhesions, and, most importantly, cytoskeletal tension^1^,^2^,^3^,^4^,^5^,^6^. One of the most cited reports to describe the effect of mechanical forces on differentiation is a study by Engler *et al.* Here, rigid substrates (>90 kPa) were shown to initiate osteogenesis in MSCs, whereas soft substrates (<11 kPa) generated neurogenesis^1^. Rigidity was shown to control these cell fates by modulating myosin contractility and the area of cell spreading. Another study has also shown that variation in spreading areas of MSCs switches their fate between osteogenic and adipogenic lineage. In this case the process is controlled by RhoA-dependent actomyosin contractility^2^. When cell spreading is constrained, cytoskeletal tension in MSCs is reduced, and this initiates adipogenesis. Extensive spreading of cells, on the other hand, permits higher cytoskeletal tension in cells and eventually leads to osteogenesis^2^,^3^. Subsequently, cell morphology has been modified with the help of micropatterned ECM geometrical cues. These cues, which modify the aspect ratio (length:breadth) and the curvature of cells, have been shown to induce a switch between osteogenesis and adipogenesis in MSCs, regardless of the soluble factors in the medium^7^. On rectangular substrates, increasing the aspect ratio led to osteogenesis^8^. At the same time, cell shapes with gentler curvature showed a more adipogenic phenotype. This study verified that focal adhesion assembly, size and myosin based contractility are the most critical determinants of these observed differentiation pathways^7^.

Similar trends of ECM mediated differentiation have repeatedly been observed under various topographical contexts^4^,^5^,^9^,^10^,^11^. For example, when MSCs were differentiated on nanogratings, focal adhesion areas were smaller and more elongated compared to those of cells grown on wider micron scale gratings. Furthermore, nanogratings produced an upregulation of neurogenic and myogenic differentiation markers. Despite these findings, inhibition of cytoskeletal contractility showed a more dominant effect on cellular differentiation than topographical control, revealing its fundamental importance to cell fate determination^5^. Additionally, ordered nanotopographical patterns resulted in diminished cell adhesion, while disordered patterns^12^,^13^,^14^ and nanoscale banding (periodicity) promoted large adhesion formations^15^,^16^. Nanoscale disordered topography significantly increased osteospecific differentiation as well^9^. Again, increased adhesion of the cells to the substrates could be directly linked to increased cell contractility^17^,^18^,^19^,^20^,^21^,^22^. Moreover, the use of specific arrangements of nanopits has also been shown to maintain multipotency of MSCs^23^,^24^.

Clearly, the biophysical components of the stem cell niche have a distinct impact on stem cell contractility and its fate. Physiologically, human MSCs characteristically inhabit the fenestrated sinusoidal capillaries made by perivascular niche, where fluid flows around the cells and generates fluid shear stresses of 0.8–3 Pa^25^. In such microenvironments, human MSCs often differentiate down an osteoblastic lineage. The literature also hints that contractile forces within human MSCs will change as the cells undergo osteogenesis^26^. Preliminary studies by Arnsdorf *et al.* suggest that Rho-dependent contractility is relevant for osteogenesis initiated by fluid shear stress^6^. However, the mechanisms of cell contractility that regulate human MSC fate in the presence of fluid shear stress remain elusive. Here, we focus on understanding the role of fluid flow on human MSC contractility and its subsequent influence on stem cell fate.

Recently, studies by Yang *et al.* have demonstrated the combinatorial effect of nanotopography and fluid shear stress in influencing MSC adhesion, spreading and migration^27^. Thus, to better understand the role of contractility in fluid shear stress mediated differentiation, we cultured human MSCs on substrates with different topographies. These regular topographies allow us to control the contractility of the human MSCs and permit us to establish the connection between ECM-influenced intracellular tension and fluid flow-regulated lineage determination process.

## Results

### Topography alters the cell spreading area

**Figure 1:**
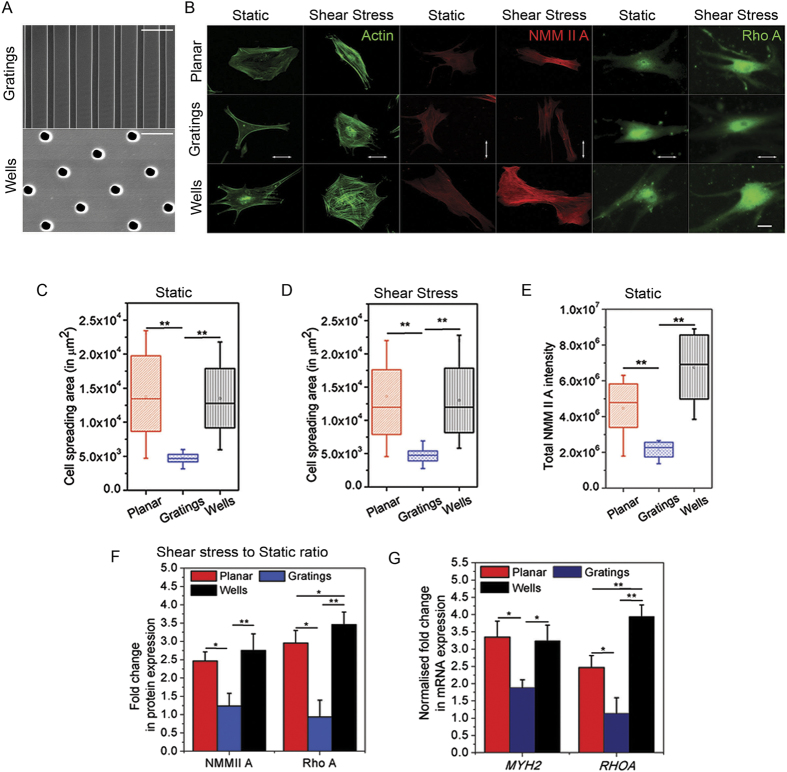
Human MSCs and change in their contractility on various topographical substrates under static condition and upon fluid shear stress exposure. (**A**) Representative images of the selected substrates (gratings and wells). (**B**) Representative images showing the spreading area of cells on planar and topographical substrates (gratings and wells) stained for phalloidin (to represent actin), NMM II A and Rho A. Scale bar: 25μm. White arrows depict grating directions. Corresponding box plot of the cell spreading area on various topographical substrates (planar, gratings and wells) in (**C**) static and (**D**) fluid shear stress, **p<0.01. 100 cells were analysed in three independent experiments. (**E**) Box plot of the protein intensity of non-muscle myosin II A (NMM II A) expression in cells cultured in static condition on various topographical substrates, **p<0.01. 80 cells were analysed in three independent experiments. (**F**) Corresponding graph showing the fold changes in the NMM II A and RhoA expression in shear experiencing cells with respect to the cells in static condition, **p<0.01, *p<0.05. (**G**) Graph showing the fold changes in *MYH2* and *RHOA* expression in shear stress experiencing cells with respect to those in static condition. mRNA expression profiles were normalized to *GAPDH* expression, **p<0.01, *p<0.05. 100 cells were analysed in three independent experiments.

Human MSCs were cultured on eight different topographies (Fig. S1A–H) and a planar substrate and their projected cell spreading area was measured (Fig. S1I). From these eight topographies, the two that produced the maximum difference in cell spreading area were chosen for further experiments: topography 1 (2 μm line, 1 μm spacing, 80 nm height) and topography 2 (1 μm diameter wells, 6.5 μm pitch, 1 μm depth) (Fig. 1A, S1A, H and I). In this paper, we will refer to the two topographies as gratings (topography 1) and wells (topography 2).

Figure 1A shows images of cells seeded on the planar substrate, gratings and wells. Cells exhibited poor spreading on the gratings, whereas they spread widely on the wells. Interestingly, the spreading area of the cells on each of the topographical substrates (gratings and wells) appeared to be on the extremes of the distribution plot of the spreading area of cells on the planar substrate (Fig. 1B,C). The spreading area of the cells did not exhibit any variation upon shear stress application (Fig. 1D). The varied spreading area of human MSCs on different topographies under static conditions could be attributed to the probable ease in which integrin attaches to the wells with respect to the gratings, which may in turn lead to preferential focal adhesion formation by the cells.

Previous studies suggest that myosin dependent contractility varies with the spreading area of cells^2^,^3^. Similarly in our study, total non-muscle myosin II A (NMM II A) expression exhibited a wide range of intensities in cells on planar substrates, and this was correlated to cell spreading area. This was in contrast to cells on wells or on gratings, which demonstrated higher, and diminished intensities, respectively (Fig. 1E). It is noteworthy that even though there is wide variation in NMM II A expression in cells on the wells, the mean distribution intensity in this condition was significantly increased compared to the mean expression in cells on either planar PDMS or gratings (Fig. 1E). Mcbeath *et al* and Gao *et al* have shown that highly spread cells are more contractile^1^,^2^. Thus, the increased NMM II A expression, which we identify in highly spread cells (on wells and planar PDMS), serves as a direct indication of increased cell contractility in this study.

Hence, the substrate topography, on which the cell is seeded, controls the intracellular contractility generated inside it by mediating alterations in cell spreading area that subsequently result in differences in cellular contractility.

Counter-intuitively, the cells on gratings did not align along the grating axis, despite this being observed by previous studies. A possible explanation for such behavior is the shallow depth of the features (80 nm), which may be insufficient to orient the cells along the grating axis^28^. Thus, for the ease of understanding, we used perpendicularly aligned gratings (with respect to the flow direction) for all experiments.

### Shear stress altered stem cell contractility on wells

To assess the change in cell contractility as mediated by fluid shear stress, human MSCs were immunostained for NMM II A and Rho A after being subjected to 48 hours of fluid flow, which generated a continuous 1 Pa shear stress. This is within the range of physiological shear stress generated during mechanical loading, which is 0.8–3 Pa^25^. Fig. 1B,E and F are the representative images, and graphs of fold change of protein expression intensity, and mRNA expression on each topography after shear stress exposure, respectively. The plots in Fig. 1F shows a 2.7-fold increase in NMM II A expression, and a 3.5-fold increase in RhoA expression, in human MSCs on wells and compared to static cells on the same topography. In contrast, human MSCs seeded on gratings exhibited a 1.2- and 0.9-fold change in NMM II A and RhoA expressions, respectively. qPCR was then used to assess the total mRNA of the *MYH2* and *RHOA* genes (Fig. 1G). This shows a 3.2- and 3.9-fold rise in *MYH2* and *RHOA* expression in human MSCs on wells, respectively. In contrast, a 1.8- and 1.1-fold change in *MYH2* and *RHOA* expression, respectively, was observed in human MSCs on gratings. These observations validate the results obtained from intensity quantification of the proteins. The change in RhoA expression in cells on wells was significantly higher than the cells on gratings or planar PDMS. Also, NMM II A expression in cells on wells showed significant increase than the cells on gratings. The quantified amplification in the expression of NMM II A and Rho A in cells on wells, directly indicates a fluid flow mediated increase in intracellular contractility in the cells.

Increased expression of NMM II A and RhoA suggests an increased engagement of actin microfilaments with motor proteins and other associated proteins, which may lead to increased cytoskeletal contractility, and possibly impinge on several cellular behaviors, including stem cell differentiation. These results suggest an immediate increase in activation of the contractile proteins upon shear stress exposure once the contractile proteins have attained a certain expression level. Cells exhibiting lower contractility have a limited response to the fluid flow in terms of contractility change.

### Increased focal adhesion alignment and adhesion area change in cells on wells

**Figure 2:**
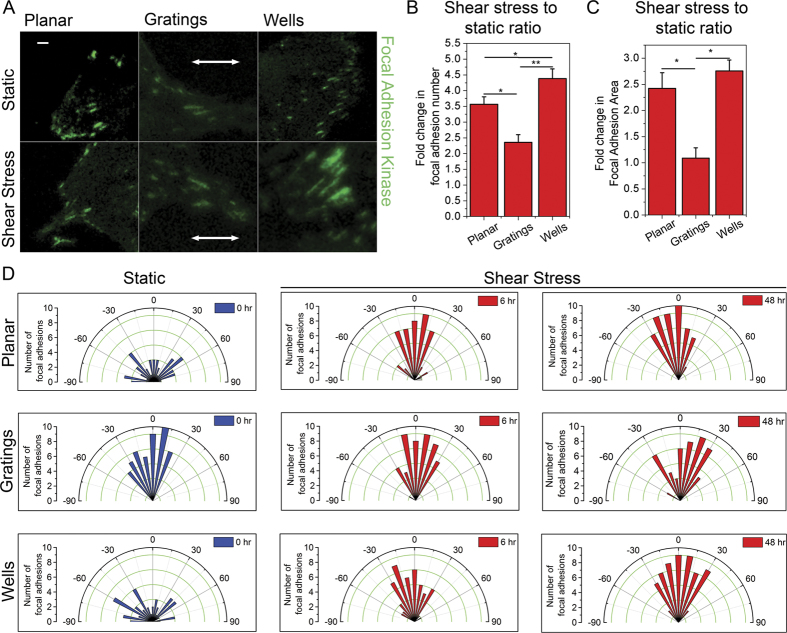
Focal adhesion changes in human MSCs experiencing fluid shear stress on various topographies. (**A**) Representative images of the focal adhesions of human MSCs, on planar PDMS, gratings and wells, stained for focal adhesion kinase (FAK) in static conditions and after shear stress application. Scale bar: 5 μm. White arrows represent the direction of the gratings. (**B**) Graph showing the fold change in the total number of focal adhesions formed in the cells exposed to fluid shear stress with respect to those in static conditions, on different topographies, **p < 0.01, *p < 0.05. 90 cells were analysed in three independent experiments. (**C**) Graph showing the fold change in focal adhesion area in human MSCs under shear stress with respect to those under static conditions on different topographies, **p < 0.01. 75 cells were analysed in three independent experiments. (**D**) Rose plot of the focal adhesion angles in human MSCs with respect to the direction of flow, with or without shear stress, on different topographies.

Forces applied during cytoskeletal reorganization alter focal adhesions^17^,^20^,^22^. Thus, after evaluating the changes in cell contractility from various topographies, we assessed the changes in focal adhesions of human MSCs upon fluid shear stress application. Fig. 2A shows human MSCs on different substrates immunostained for focal adhesion kinase (FAK). Sizes of single focal adhesion complexes were measured. Under static conditions, the size of focal adhesions in human MSCs on gratings is larger than those of human MSCs on wells. However, Fig. 2C suggests that post fluid flow application, there was a 2.5-fold increase (statistically significant) in the size of focal adhesions in cells on wells. This was in contrast to the lack of significant change in the size of focal adhesions in cells on gratings. At the same time, Fig. 2B shows that the total number of focal adhesions in human MSCs on wells was double that of cells on gratings (statistically significant). Furthermore, this increase in focal adhesion number in cells on wells was significantly higher than that of cells on planar substrates. Prior to fluid flow application, focal adhesions were randomly aligned in human MSCs on the wells whereas on gratings, they were aligned in one direction. However, after fluid flow exposure, focal adhesions on wells aligned along the direction of the flow while the alignment of focal adhesions remained unchanged in the cells on gratings (Fig. 2D).

These changes in focal adhesions hint that the surface receptors in MSCs sense the applied mechanical stress and this induces actomyosin cytoskeleton reorganization. This subsequently leads to increase in focal adhesion number and also causes focal adhesion alignment to follow the direction of flow. We also speculate that an increase in cell contractility reinforces the focal adhesions and causes increase in their sizes. Thus, it is likely that in cells seeded onto the wells, cell contractility is high enough to drive downstream signaling and focal adhesion alterations. However, on gratings, since the contractility is considerably low, cells are unable to generate enough intracellular tension and cannot respond to the forces acting on them and are thus unable to activate osteogenic pathways during the 48 hours of shear stress application.

### Cells on isotropic topography followed an osteogenic lineage upon shear stress exposure

**Figure 3:**
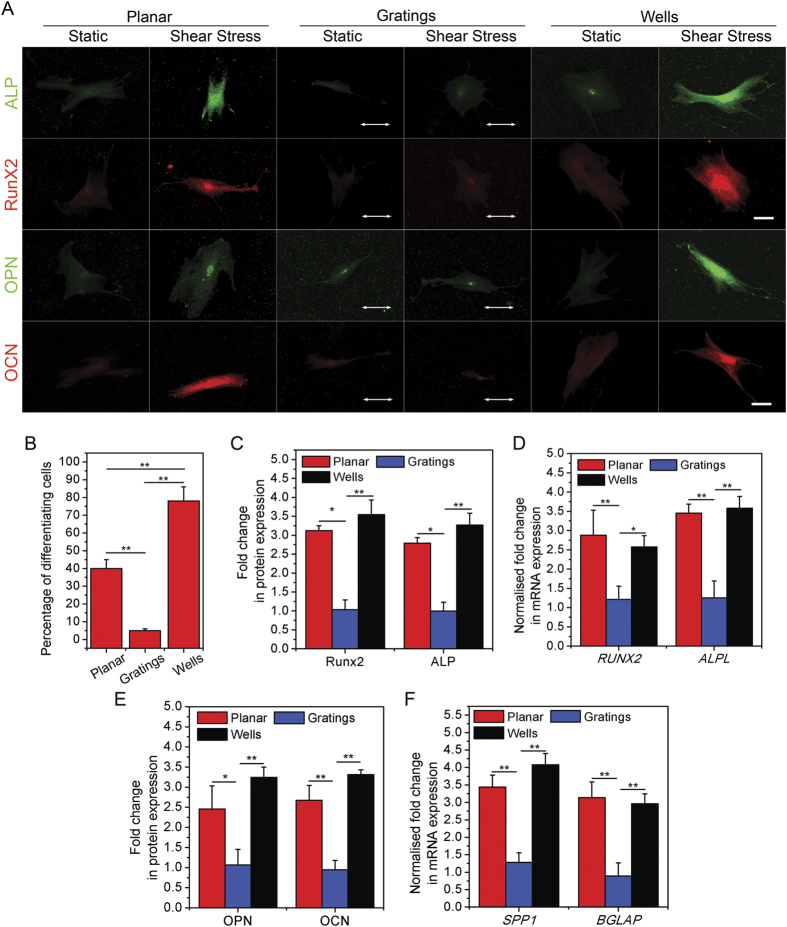
Human MSC differentiation upon fluid flow exposure on different topographical substrates. (**A**) Representative images of cells stained for early osteogenic markers (ALP and Runx2) and late osteogenic markers (OPN and OCN) in human MSCs experiencing fluid shear stress, or with static conditions, while seeded on gratings and wells. White arrows depict grating directions. Scale: 10 μm. (**B**) Graph depicting the percentage of human MSCs expressing osteogenic markers (Runx2 and OPN) after 48 hours of fluid flow exposure on different substrate topographies, **p < 0.01. 200 cells were analysed in three independent experiments. (**C**) Corresponding graph showing the fold change in early osteogenic marker intensity in cells experiencing shear stress, with respect to those under static conditions, **p < 0.01, *p < 0.05. 90 cells were analysed in three independent experiments. (**D**) Graph showing the fold change in the expression of *ALPL* and *RUNX2* in human MSCs (normalized to *GAPDH* expression) undergoing shear stress with respect to those in static conditions. Cells were cultured on planar PDMS, gratings or wells, **p < 0.01, *p < 0.05. (**E**) Corresponding graph showing the fold change in protein expression in cells experiencing fluid shear stress with respect to those in static conditions, when seeded on different topographies, **p < 0.01, *p < 0.05. 75 cells were analysed in three independent experiments. (**F**) Graph showing the fold change in *SPP1* and *BGLAP* expression in human MSCs (normalized to *GAPDH* expression) undergoing shear stress with respect to those in static conditions, while being cultured on planar PDMS, gratings and wells, **p < 0.01.

In light of the effects fluid shear stress had on the contractility of human MSCs, as well as focal adhesion changes, it is important to evaluate how fluid flow can direct lineage determination of human MSCs on different topographical substrates. Osteogenic differentiation medium was used in these experiments. To better understand differentiation in the human MSCs, the cells were stained for early (Runx2 and alkaline phosphatase (ALP)) and late (osteopontin (OPN) and osteocalcin (OCN)) osteogenic markers after being exposed to fluid flow for 48 hrs. Fig. 3A, C and D depict the fold change in the intensity of protein expression and genetic expressions of early markers (Runx2 and ALP) of osteogenic differentiation. On wells, after shear stress exposure, the expression of both Runx2 and ALP increased 3.5-fold and 3.2-fold, respectively, whereas on gratings, there was no considerable change in their expression (Fig. 3C). The amount of mRNA transcribed by the cells on both topographies validated the results obtained from quantification of protein intensities. As can be seen in Fig. 3D, after fluid flow, quantification of mRNA levels from cells on wells showed an increase of 2.6-fold and 3.5-fold for *RUNX2* and *ALPL* respectively. In cells on gratings, there was no significant change in expression level of either gene.

Figure 3A, E and F show changes in the expression of markers of late stage osteogenesis (OPN and OCN) in cells on either topography. After shear stress application, expression of OPN and OCN in cells on wells showed 3.2-fold and 3.3-fold augmentation, respectively (Fig. 3E). However, in cells on gratings, the expression of these late stage marker proteins remained unchanged. Similarly, the expression of *SPP1* and *BGLAP* mRNA (mRNA corresponding to OPN and OCN proteins) in cells on wells increased 4-fold and 2.9-fold upon shear stress exposure, respectively, whereas on gratings, the mRNA levels of the osteogenic markers remained unchanged under fluid shear stress (Fig. 3F).

The percentage of human MSCs undergoing differentiation on a planar substrate, and on wells, was also measured through the quantification of Runx2 and osteopontin expression. This data is plotted in Fig. 3B, and as can be seen, only 40% of the cells underwent differentiation on planar substrate following fluid flow. However, this was amplified to 70% in cells on wells. In the static condition, hMSCs exhibited 74% osteogenic differentiation on wells, and 35% and 50% differentiation on gratings and planar substrates, respectively, in 21 days (Fig. S3D).

These results suggest that after 48 hours of shear stress exposure, human MSCs undergo osteogenesis on wells, however there is no conclusive evidence of human MSC differentiation in cells on gratings.

### Cells on gratings remained multipotent despite the use of osteogenic biochemical cues

**Figure 4:**
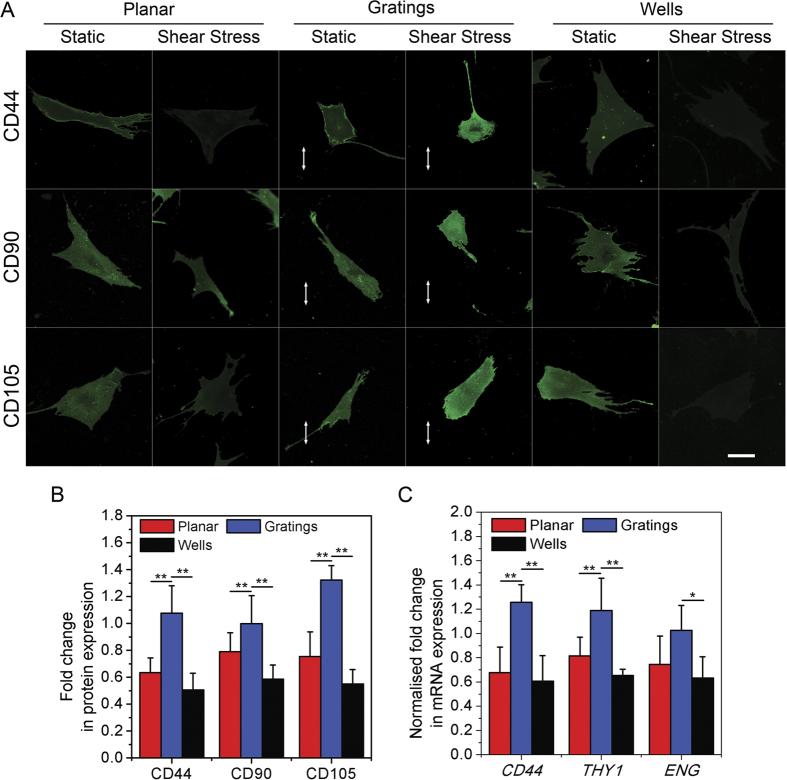
Human MSC multipotency after fluid flow exposure on different topographical substrates. (**A**) Representative images of cells stained for the multipotency markers, CD44, CD90 and CD105, in cells undergoing fluid shear stress on either planar PDMS, wells or gratings White arrows depict grating directions. Scale: 10 μm. (**B**) Corresponding graph showing the fold change in the intensity of the multipotency markers, CD44, CD90 and CD105, in cells under shear stress, or static conditions, **p < 0.01, *p < 0.05. 75 cells were analysed in three independent experiments. (**C**) Graph showing the fold change in *CD44, THY1* and *ENG* expression in human MSCs (normalized to *GAPDH* expression) undergoing shear stress with respect to those in static conditions. Cells were cultured on either planar PDMS, gratings or wells, **p < 0.01, *p < 0.05.

Having established the fluid shear stress mediated fate of human MSCs on wells, we proceeded to ascertain the fate of these cells while experiencing fluid flow on gratings. Thus, human MSCs were seeded onto different topographies and stained for the multipotency markers CD44, CD90 and CD105 after being exposed to fluid shear stress for 48 hours (Fig. 4). The plot in Fig. 4B shows that upon fluid flow application, multipotency of human MSCs was reduced when the cells were on wells, but remained unaltered when cells were on gratings. mRNA expression of the markers, as plotted in Fig. 4C, also supported the protein expression results. This suggests that human MSCs remain multipotent on gratings even after application of an external stress. This is in contrast to when the cells are on gratings, where they begin the process of differentiation as soon as they are subjected to an external force. This could be explained by an enhanced differentiation potential of human MSCs on wells and augmented multipotency of human MSCs on gratings.

### Effect of actomyosin and focal adhesion inhibitors

**Figure 5:**
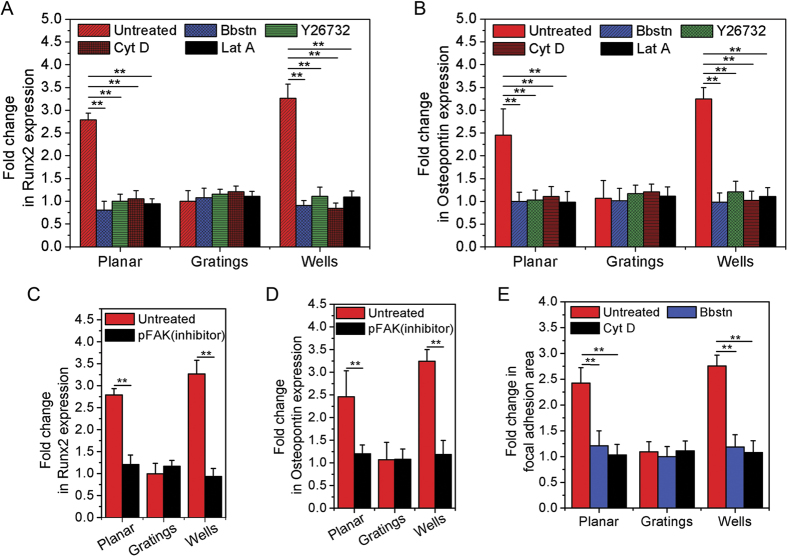
Effects of inhibitors on fluid shear stress mediated human MSC fate on different topographies. Graph showing the fold change in (**A**) Runx2 and (**B**) OPN expression in human MSCs undergoing fluid shear stress exposure on different topographies in the presence of actomyosin inhibitors (blebbistatin, Y26732, cytochalasin D and latrunculin A) with respect to those in static condition, **p < 0.01. 100 cells were analysed in three independent experiments. Graph showing the fold change in (**C**) Runx2 and (**D**) OPN expression in human MSCs while experiencing fluid shear stress on different topographies in the presence of pFAK inhibitor (PF573228), **p < 0.01. 75 cells were analysed in three independent experiments. (**E**) Graph showing the fold change in focal adhesion area in human MSCs upon shear exposure with respect to cells under static conditions, but in the presence of actomyosin inhibitors (blebbistatin and cytochalasin D), **p < 0.01. 90 cells were analysed on three independent experiments.

To elucidate the role of actomyosin contractility in fluid shear stress directed human MSC osteogenesis, cells were treated with several cytoskeletal contractility inhibitors. Actomyosin contractility was inhibited using blebbistatin (myosin II inhibitor; Bbstn), Y-27632 (ROCK inhibitor), cytochalasin D (actin inhibitor; Cyt D) and latrunculin A (actin inhibitor; Lat A). With actomyosin contractility inhibited, the cells were then subjected to fluid shear stress, and the expression levels of Runx2 and osteopontin were measured and plotted (Fig. 5). As opposed to untreated cells, the drug-treated cells did not express osteogenic markers when exposed to fluid flow, and this was regardless of whether the cells were seeded onto wells or gratings (Fig. 5 (A,B)).

Likewise, when FAK phosphorylation was inhibited using PF573228 (Y397-pFAK inhibitor), the expression of Runx2 or osteopontin was not altered by fluid shear stress, and again this was independent of whether the cells were seeded onto wells or gratings (Fig. 5C,D). This could be explained by Fig. 5E which shows that under actomyosin inhibition, focal adhesion area remained unaltered. These results indicate that human MSCs with impaired intracellular contractility or FAK phosphorylation did not undergo osteogenic differentiation when fluid shear stress was applied.

The augmented differentiation of cells on wells in the presence of fluid shear stress could be due to increase in intracellular contractility. The augmentation in cell contractility may indicate that the cell is preparing to initiate osteogenic differentiation, and fluid shear stress, an external force serves to drive these pre-stressed human MSCs down the osteoblastic lineage. This observation iterates that optimal level of contractile proteins are required before an external force can initiate the process of differentiation in these cells. These results also emphasize the pivotal role played by the actomyosin cytoskeleton in the transmission of fluid shear forces detected at the surface of the cell to the other cell processes which subsequently direct the fate of the cells.

## Discussion

Human MSCs typically grow in the bone marrow within a niche formed by fenestrated sinusoidal capillaries^29^ that are, on average, 100 nm in diameter^30^. Typically, endothelial cells of the sinusoidal walls form these fenestrations^31^. This means that human MSCs inherently reside in regular topographies and the importance of topographical features in influencing MSC growth and fate has been highlighted over the last 30 years through *in vitro* studies^4^,^5^,^10^,^11^,^24^,^25^,^26^,^27^. From such studies, a better understanding of the effect of the cellular microenvironment can be attained which will thus aid in developing better tissue engineering platforms for cell therapy.

In this study, we showed that when human MSCs are seeded onto ‘well’ topography, they spread widely and generate higher contractility. In contrast, when they were seeded onto the ‘gratings’ topography, they were poorly spread on and had low contractility. Remarkably, the application of fluid flow to the cells, had no effect on the cell area or spread, regardless of the topography the cells were on.

The initial difference in the cell spreading area could be attributed to differences in cell contractility, as seen previously^2^,^7^. Interestingly, upon fluid flow exposure, the contractility of the cells on wells augmented while the contractility of the cells on gratings remained the same. Changes in cell contractility is known to elicit stem cell differentiation^6^ and the relevance of intracellular tension has also been cited to maintain the multipotency of MSCs on topographies^23^. Thus, it is likely that the cells on gratings remain multipotent and hence do not exhibit contractility change even after being subjected to shear stress.

Furthermore, focal adhesions connect the actomyosin cytoskeleton to the ECM and are thus proposed to be important in the cells ability to sense topography^5^,^32^. In this study, we observed an increase in focal adhesion number in cells seeded on wells when they were exposed to fluid flow. The focal adhesions were also aligned in the direction of the fluid flow. Notably, the focal adhesions in cells on gratings did not exhibit any variation after shear stress exposure.

The differences observed in focal adhesions following fluid flow could be attributed to the topographies beneath the cells and the interaction of the focal adhesions with them. We hypothesize that the topographical information is transduced into lineage determining signaling cascades via actin cytoskeleton, and these signals then modulate the formation and maturation of focal adhesions^17^,^18^,^19^,^20^,^21^,^22^. Such a phenomenon has been seen in human MSCs cultured on nanogratings, where the focal adhesions aligned along the grating axis, and the cytoskeleton aided in translating the information from the focal adhesion to the cell nucleus^5^. Similarly, we propose that the changes in focal adhesion number and size could also influence the regulation of gene expression in human MSCs. Our observations that fluid flow mediates change in focal adhesions supports earlier findings that suggest mechanical stresses can alter focal adhesions^33^.

**Figure 6:**
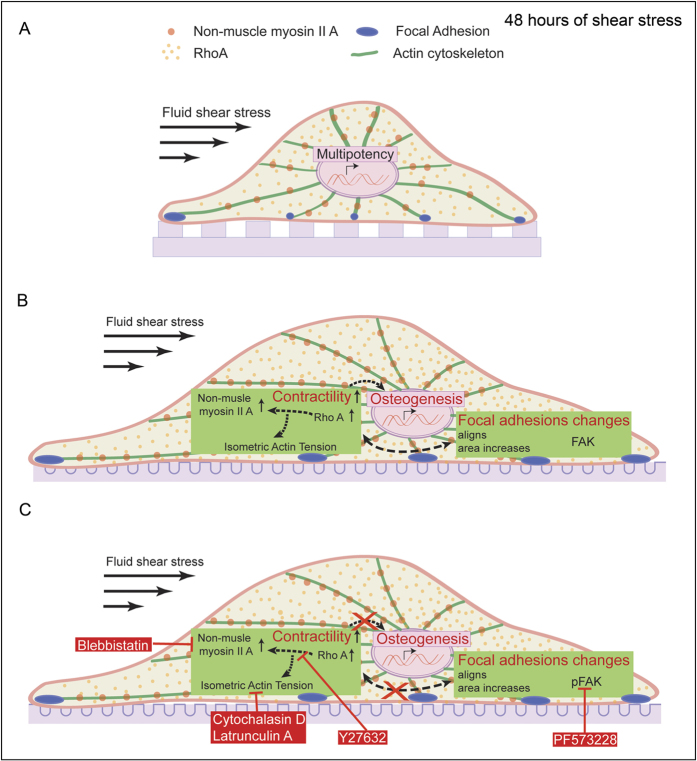
FSS mediated fate in human MSCs in 48 hours of flow induction. (**A**) On gratings, 48 hours of fluid shear stress had no effect on human MSCs and the cells remained multipotent. (**B**) On wells, fluid shear stress induced an increase in cellular contractility, leading to focal adhesion changes and osteogenesis. (**C**) Pharmacological antagonists of FAK phosphorylation or actomyosin contractility inhibit fluid shear stress directed osteogenesis in human MSCs on wells.

Differentiation of human MSCs can be mediated by both shear stress, and ECM topographies, and in these cases, the differentiation are regulated by cytoskeletal contractility^5^,^6^. For this reason we hypothesize that the human MSCs exhibiting increased contractility after being exposed to fluid flow, will undergo differentiation, whereas those with low contractility will remain undifferentiated. Indeed after 48 hours of fluid flow application, cells on wells were found to have augmented osteogenic markers (ALP, Runx2, OPN and OCN). This was observed both at the genetic and at the protein levels (Fig. 6B). However, within 48 hours of shear stress exposure, cells on gratings remained positive for multipotency markers, CD44, CD90 and CD105 (Fig. 6A). When actomyosin contractility or phosphorylation of FAK was impaired using specific inhibitors, hMSCs did not undergo osteogenic differentiation, even after being exposed to external shear stress (Fig. 6C). These results emphasize the pivotal role played by actomyosin contractility in fluid shear stress mediated stem cell differentiation. This also hints at the relevance of basal cellular contractile forces in the process of fate determination, with stem cells requiring a certain level of contractility to undergo osteogenesis. Cells with a lower contractility tend to remain in a quiescent state and maintain their multipotency, even after extracellular forces are applied. It is possible that fluid flow forces alone are unable to recruit the contractile motors and proteins that are required by the cell to generate forces need to trigger the signaling cascades pertaining to osteogenesis. Differentiation of human MSCs on wells could be a result of the increased spreading area and contractility of the cells. We speculate that the well-shaped substrates provide an easy attachment of integrins to the ECM proteins which allows increased contractility of the cells. We also believe that upon shear stress application, the ease of focal adhesion formation in cells on wells promotes the increment in focal adhesion size and number in cells adhered to them.

Together, these results indicate that lineage determination may be decided at the level of cell-substrate interaction. Furthermore, the contractility of the human MSCs, which will be determined by the mechanical properties of the substrate, cannot be altered by external shear forces until the cells have reached a certain contractile potential. The results here also support the idea that focal adhesions act as the mechanosensors to sense the topography of the substrate, and subsequently influence the contractile mechanisms of the cells. The modulation of the actomyosin cytoskeleton, which can result from fluid shear forces received by the focal adhesions, attenuates cell contractility, which is controlled by the ECM topographical signals. Together these factors regulate the fate of the human MSCs. The findings here suggest that stem cell contractility, as determined by the ECM topography, is a more important factor in determining the cell fate than the application of external force. However, we still recognize the relevance of increment in the contractile proteins caused by fluid forces, which eventually induces osteogenesis. Nevertheless, the short duration of these experiments also opens up a discussion on the possibility of delayed differentiation in cells on gratings. Thus, longer shear stress exposure experiments must be performed to better understand lineage determination of human MSCs on gratings.

## Conclusion

In summary, intracellular tension in human MSCs on wells increases upon exposure to fluid flow forces. These intracellular changes in actomyosin filaments activate osteogenic differentiation-related genes to initiate osteogenesis. Notably, for external forces to initiate osteogenesis in human MSCs, the cells need to generate higher contractility prior to the application of force. When the level of cellular contractility remains low, the human MSCs will retain their multipotency. This leads us to hypothesize that if stem cells are to maintain their multipotency, they must limit the level of intracellular contractility to a point that allows cell proliferation, without causing the cells to become rounded, eventually leading to adipogenesis. Thus, an optimal intracellular contractility threshold needs to be met in order to trigger crucial metabolic and biochemical signaling pathways that facilitate active differentiation. Hence, cellular contractility and tension is a relevant modular mechanism for the fate of human MSCs.

From our study, it is quite evident that substrate topography guided cell responses to fluid shear stress vary with the topography of the substrate and must be considered for optimal control of stem-cell expansion and differentiation. Since human MSCs are used for tissue engineering purposes, the results obtained in this study will potentially be useful in designing scaffolds and materials for stem cell culture required for tissue engineering therapies.

## Methods and Materials

### Substrate and channel fabrication

Eight distinct topographies were chosen (Fig. S1A–H):

- 2 μm line, 1 μm spacing, 80 nm height
- 1 μm line, 2 μm spacing, 120 nm height
- 250 nm line, 250 nm spacing, 250 nm height
- 250 nm line, 250 nm spacing, 110 nm height
- 460 nm line, 70 nm spacing, 40 nm height
- 2 μm diameter pillars, 12 μm pitch, 2 μm height
- 500 nm diameter pillars, 10 μm pitch, 500 nm height
- 1 μm diameter Wells, 6.5 μm Pitch, 1 μm Depth

Topography A and H exhibited the maximum difference in the cell spreading area of human MSCs, and were therefore chosen for rest of the experiments. For immunostaining experiments, these topographies were stitched adjacently. For mRNA quantification, same topographies were stitched together for making channels. Besides the static controls of both topographies, planar PDMS (Polydimethyl siloxane; Sylgard^®^ 184, Dow Corning) substrates were used as internal controls in each experiment.

### Topographical Microfluidic Chamber

A topographical microfluidic chamber was fabricated by double casting of PDMS. Prior to patterned PDMS fabrication, the polycarbonate wafer was vacuum silanised using Trichloro (1H, 1H, 2H, 2H-perfluorooctyl) silane (Sigma-Aldrich Co. LLC) and then incubated in surfactant (0.01% Triton X-100, Sigma-Aldrich Co. LLC) for 1 min. A homogeneous mixture curing agent and PDMS elastomer (1:10 ratio) was degassed, poured on the wafer and heated for curing at 80 °C for 2 hours. Cured PDMS was then peeled from the wafer, vacuum silanized and subsequently used as a PDMS negative wafer to fabricate PDMS topographical substrates. Uncured PDMS was spin-coated on glass coverslips using a spin coater and silanized PDMS wafers were placed upon them and then cured at 80 °C for 2 hours. Glass coverslips with spin-coated PDMS containing topographies (with elasticity of ∼10 MPa) were plasma cleaned and then attached to pre-fabricated PDMS microchannels (with a method similar to making PDMS mold from the polycarbonate wafers) to obtain a parallel plate microfluidic chamber (50 mm long, 2 mm wide and 120 μm high) (Fig. S2A). Gratings were in perpendicular orientation to the intended direction of fluid flow.

### Cell Culture and Fluid Flow conditions

Human MSCs (Lonza Inc.) were cultured in human MSC growth medium (Lonza Inc.) supplemented with 1% penicillin/streptomycin (Invitrogen). Experiments were performed using passages 1–5 of cells. Channels were coated with 50 μg/ml Fibronectin (Sigma-Aldrich Co. LLC) for 1 hour, washed with PBS (1X; Phosphate Buffer Saline) and then 1000 cells/cm^2^ were seeded inside the channels.

For flow experiments, osteogenic medium (Dulbecco’s Minimum Essential Medium (DMEM)), added with 10% fetal bovine serum (Invitrogen), 1% penicillin/streptomycin (Invitrogen), 5 mM β- glycerophosphoric acid (Sigma-Aldrich Co. LLC), 10^−2^ μM Dexamethasone (Sigma-Aldrich Co. LLC), and 50 μg/ml L-ascorbic acid (Sigma-Aldrich) was used.

Pa fluid shear stress was applied on cells seeded in these topographical microfluidic channels after calibrating the flow conditions. A peristaltic pump (Model P720; Instech) was used to apply laminar fluid flow on cells. A reservoir of culture medium was attached to the peristaltic pump which was attached to a bubble trap and dampener on the other end. This entire setup was connected to the microfluidic chamber inlet. The chamber outlet was connected to the reservoir to form a closed loop of the flowing fluid. This microfluidic setup (except peristaltic pump) was placed inside an incubator (37 °C and 5% CO_2_) (Fig. S2B).

### Immunocytochemistry, confocal imaging and quantification

After fluid flow exposure, human MSCs were fixed with 4% paraformaldehyde (Sigma-Aldrich Co. LLC), permeabilized with 0.3% Triton X-100 (Sigma-Aldrich Co. LLC) and blocked using 1% bovine serum albumin (BSA; Sigma-Aldrich Co. LLC) in 1X PBS. Once blocked, human MSCs were incubated with the primary antibody of interest in 1% BSA overnight. The primary antibodies used were Anti-focal Adhesion Kinase (Cell Signaling Technology) at 1:500, Anti-Runx2 (SantaCruz Biotechnology, Inc.) at 1:200, Anti-osteopontin (SantaCruz Biotechnology, Inc.) at 1:200, Anti-osteocalcin (SantaCruz Biotechnology, Inc.) at 1:200, Anti-CD44 (Abcam^®^) at 1:500, Anti-CD90 (Abcam^®^) at 1:500 and Anti-CD105 (Abcam^®^) at 1:500. Primary antibodies were then incubated with fluorescent dye tagged-secondary antibody (Alexa Flour^®^; Invitrogen^™^, Life Technologies) in 1% BSA for 1 hour. Cells were not permeabilised for multipotency marker staining.

Alkaline Phosphate (ALP) staining was performed with 1X ALP live stain (Invitrogen^™^, Life Technologies) for 20 mins.

Several positions in the sample were imaged randomly on a confocal microscope (Nikon Confocal A1R). In the obtained images, cells were outlined as region of interest and the integrated density was measured for the z-projection of the z-stack of each cell. Product of cell area and mean fluorescence of the background readings were subtracted from the measured integrated density of the cell to calculate the corrected total cell fluorescence. Graphs were plotted to show the fold change of the corrected total cell fluorescence for each protein in cells subjected to shear stress, with respect to the corrected total cell fluorescence of that protein in cells in static conditions.

### mRNA expression profiles

After shear stress exposure, total RNA was isolated from the cells using RNeasy mini kit (Qiagen) and reverse-transcribed with iScript^™^ reverse transcription supermix for RT-qPCR (Bio-Rad). Quantitative PCR (qPCR) was performed using SsoFast^™^ EvaGreen^®^ Supermix (Bio-Rad) on a Bio-Rad CFX96^™^ Real-Time PCR Systems. Primers and probe sets for *MYH2* (non-muscle myosin heavy chain), *RHOA* (RhoA), *ALPL* (alkaline phosphatase), *RUNX2* (runt-related transcription factor 2), *SPP1* (osteopontin), *BGLAP* (osteocalcin), *CD44* (CD44), *THY1* (CD90), *ENG* (CD105) and *GAPDH* (GAPDH) were purchased from AIT Biotech (Table S1). Each primer expression was normalised by *GAPDH* expression.

### Inhibitor drug treatments

To perform pharmacological drug inhibition, human MSCs were treated before fluid flow application with 25 μM Blebbistatin (Sigma-Aldrich) for 30 mins, 5 μM Y-27632 (Sigma-Aldrich) for 10 mins, 100 nM Cytochalasin D (Sigma-Aldrich) for 30 mins, 5 μM Latrunculin A (Sigma-Aldrich) for 1 hour and 100 nM PF573228 (Tocris Bioscience) for 30 mins. Post drug treatment, cells were exposed to fluid flow with medium supplemented with the respective drugs. Graphs were plotted as a ratio of protein expression of the cells experiencing fluid flow under the effect of drug to that of protein expression of the static cells cultured in the presence of drug.

### Statistics

Student unpaired t-test performed for statistical analyses. Data presented here represent the mean ± SD for independent experiments (n = 3). At p < 0.01 or p < 0.05, data was considered significant^30^,^31^,^32^,^33^.

## Additional Information

**How to cite this article**: Sonam, S. *et al.* Cell contractility arising from topography and shear flow determines human mesenchymal stem cell fate. *Sci. Rep.* **6**, 20415; doi: 10.1038/srep20415 (2016).

## Supplementary Material

Supplementary Information
