# Supplementary material for: Cell contractility arising from topography and shear flow determines human mesenchymal stem cell fate
Source: Sci Rep. 2016 Feb 16;6:20415. doi: 10.1038/srep20415 (PMC4754642; doi:10.1038/srep20415)
Supplement: Supplementary Information [file srep20415-s1.pdf]

Cell contractility arising from topography and shear flow determines human mesenchymal stem cell fate

Surabhi Sonam[1, 2], Sharvari R. Sathe[1], Evelyn K.F. Yim[1, 2, 3], Michael P. Sheetz[1, 4], Chwee Teck Lim*[,1, 2, 5]

[1]Mechanobiology Institute, National University of Singapore, 117411, Singapore

[2]Department of Biomedical Engineering, National University of Singapore, 117583, Singapore

[3]Department of Surgery, National University of Singapore, 119228, Singapore

[4]Department of Biological Sciences, Columbia University, New York, 10027, USA

[5]Department of Mechanical Engineering, National University of Singapore, 117575, Singapore

*****ctlim@nus.edu.sg

Supplementary Figures

Supplementary figure S1. Substrate topographies

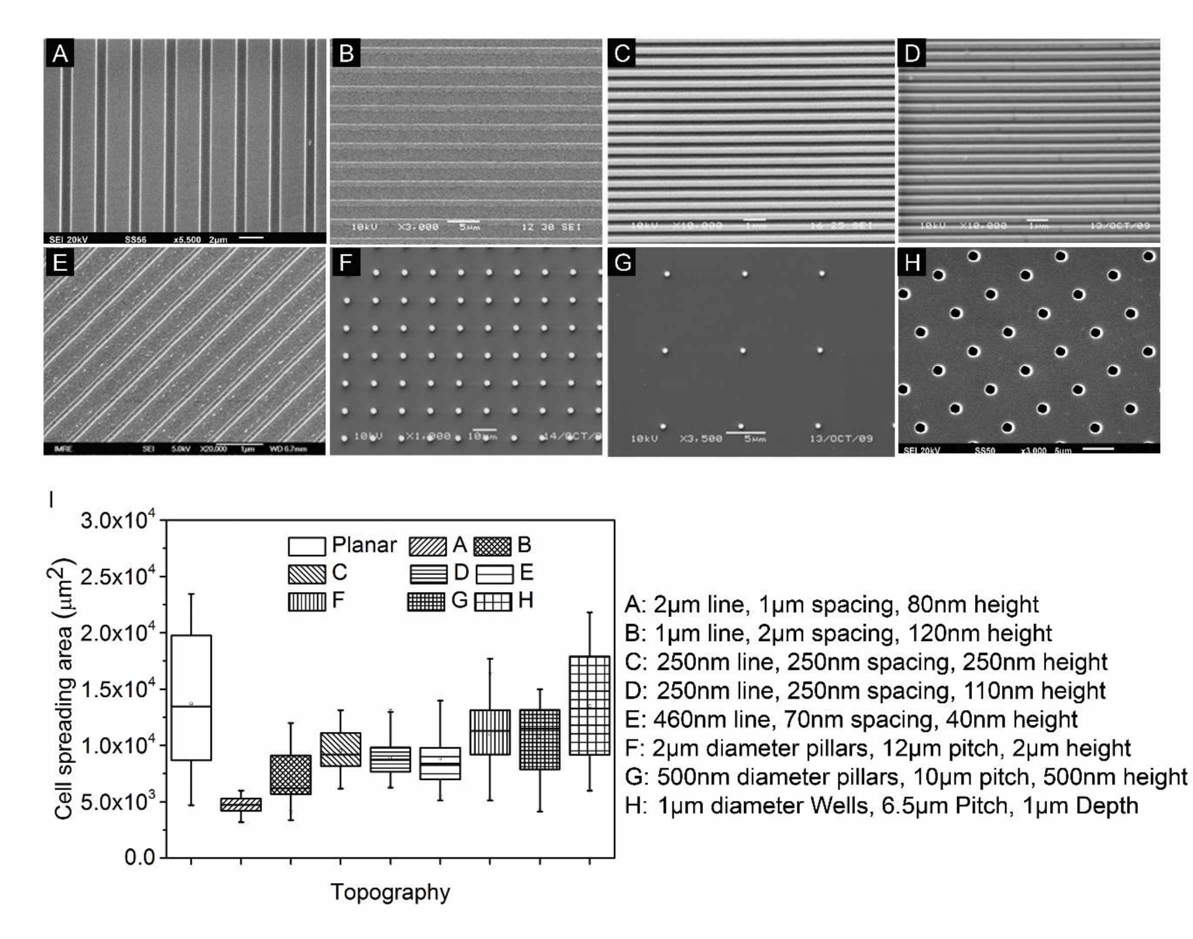

Supplementary figure S1| Scanning electron micrographs of A) 2µm line, 1µm spacing, 80nm height, Scale: 2µm, B) 1µm line, 2µm spacing, 120nm height, Scale: 5µm, C) 250nm line, 250nm spacing, 250nm height, Scale: 1µm, D) 250nm line, 250nm spacing, 110nm height, Scale: 1µm, E) 460nm line, 70nm spacing, 40nm height, Scale: 1µm, F) 2µm diameter pillars, 12µm pitch, 2µm height, Scale: 10µm, G) 500nm diameter pillars, 10µm pitch, 500nm height, Scale: 5µm, H) 1µm diameter Wells, 6.5µm Pitch, 1µm Depth, Scale: 5µm, I) Box plot of spreading area of cells on each of the topographies

Supplementary figure S2. Topographical microfluidic channel

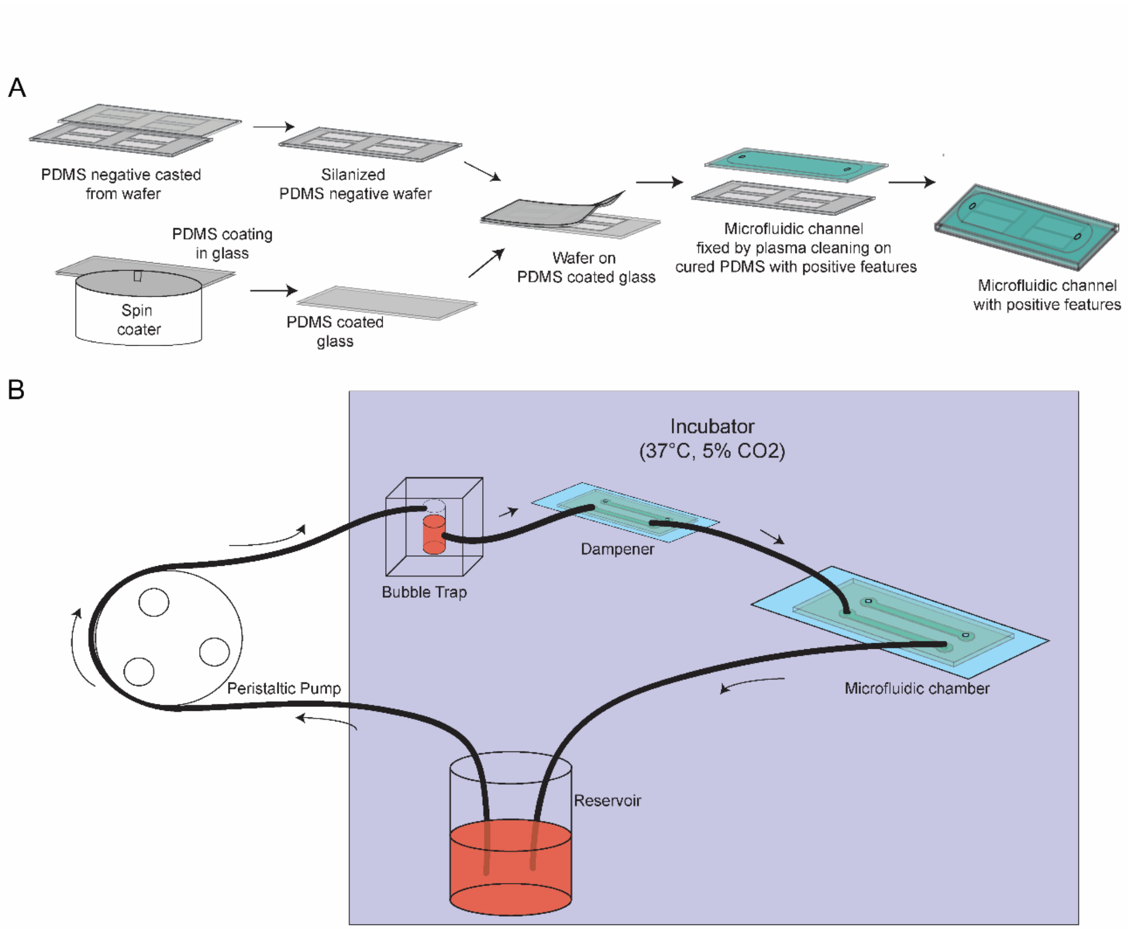

Supplementary figure S2| A. Illustration of process of fabrication of microfluidic channel with topographies. B. Illustration of fluid flow setup.

Supplementary figure S3. Calibration of fluid flow regime formed on different topographies

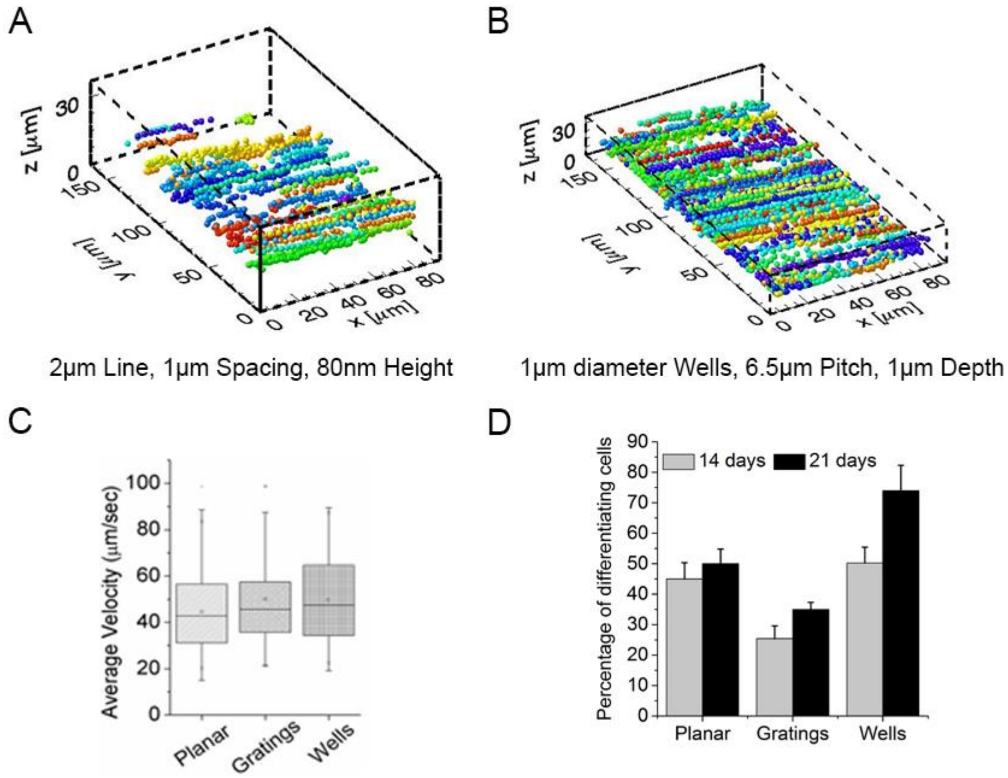

Supplementary figure S3| Tracks of bead movement with flowing fluid over topographies seeded with human MSCs. Collective tracks of individual beads (each bead represented by a color) flowing over human MSCs seeded over (A) gratings and (B) wells plotted in a 3D graph. C. Box plot of average velocity of the flowing beads. 50 beads were tracked in 3 independent experiments. The gratings were aligned perpendicular to the flow direction. There is no statistical difference ($p>0.05$). D. Plot of percentage human MSCs differentiating on planar substrates, gratings and wells.

Supplementary figure Table S1. List of primer sequences used for qPCR

Gene	Forward Primer Sequence	Reverse Primer Sequence
GAPDH	CTTTGTCAAGCTCATTTCCTGG	TCTTCCTCTTGTGCTCTTGC
MYH2	CAGACCAAAGAACAGGCAGA	TCGCATCAATAAAGCTCTGG
RHOA	GGGAGCTAGCCAAGATGAAG	TGGAGTGTTCAGCAAAGACC
ALPL	GATGTGGAGTATGAGAGTGACG	GGTCAAGGGTCAGGAGTTC
RUNX2	TTCACCTTGACCATAACCGTC	GGCGGTCAGAGAACAAACTAG
SPP1	AGGCTGATTCTGGAAGTTCTG	CTTACTTGGAAGGGTCTGTGG
BGLAP	TGACGAGTTGGCTGACCA	AGGGTGCCTGGAGAGGAG
CD44	ACCCAAATCATTCTGAAGGC	ACCTTCATCCCAGTGACCTC

THY1	CCTAGTGGACCAGAGCCTTC	CAGTTCACCCATCCAGTACG
ENG	CTCTCTGGGCCTTGAGTTTC	ACCGTCTCTGGGTTCAAATC
